# p21 in Cancer Research

**DOI:** 10.3390/cancers11081178

**Published:** 2019-08-14

**Authors:** Bahar Shamloo, Sinem Usluer

**Affiliations:** 1 Department of Oncological Sciences, Huntsman Cancer Institute, University of Utah, Salt Lake City, UT 84112, USA; 2 Department of Molecular Biology & Biochemistry, Gottfried Schatz Research Center for Cell Signaling, Metabolism and Aging, Medical University of Graz, 8010 Graz, Austria

**Keywords:** p21, cancer, therapeutic approach, p53, gene editing

## Abstract

p21 functions as a cell cycle inhibitor and anti-proliferative effector in normal cells, and is dysregulated in some cancers. Earlier observations on p21 knockout models emphasized the role of this protein in cell cycle arrest under the p53 transcription factor activity. Although tumor-suppressor function of p21 is the most studied aspect of this protein in cancer, the role of p21 in phenotypic plasticity and its oncogenic/anti-apoptotic function, depending on p21 subcellular localization and p53 status, have been under scrutiny recently. Basic science and translational studies use precision gene editing to manipulate p21 itself, and proteins that interact with it; these studies have led to regulatory/functional/drug sensitivity discoveries as well as therapeutic approaches in cancer field. In this review, we will focus on targeting p21 in cancer research and its potential in providing novel therapies.

## 1. p21 and Cancer

### 1.1. p21 in Early Days

Imbalance between cell proliferation and cell death (apoptosis) leads to tumorigenesis. p21, a well-established cyclin-dependent kinase (cdk) inhibitor, was found to play an important role in controlling cell cycle progression [1]. In 1994, p21 (also known as wildtype activating factor-1/cyclin-dependent kinase inhibitory protein-1 or WAF1/CIP1) was introduced as a tumor suppressor in brain, lung, and colon cancer cells; it was shown that p21 induces tumor growth suppression through wild type p53 activity [2]. Mousses et al. reported some evidence that indicated the link between tumor development and p21 protein alteration [3]. While p21 alteration was not found to be responsible for cancer development in certain cancer types, such as ovarian or breast cancer [4,5], there were evidence supporting the reverse scenario in other tumor types such as thyroid or endometrial carcinoma [6,7]. An early study on non-small cell lung carcinoma showed that p21 is overexpressed in well-differentiated tumors [8]. p21 has been mostly associated with p53 protein regarding its cell cycle arrest role; there are studies that showed p53-independent pathways leading to p21 induction at early years of its discovery [9]. In one of these early studies, p21 was shown as an immediate-early gene, with transcription peak at 2 hours in the presence of certain growth factor, independent of p53 protein [9]. These studies were directed towards the fact that through p21 induction in p53-null cancer cells, G1 checkpoint can be restored and cell cycle arrest could be activated [10]. p21 was found to be associated with cellular sensitivity to Transforming Growth Factor-beta (TGF-beta) at the same time, exploring where p21 stands in cancer development [11], considering TGF-beta role in premalignant state, malignant progression, invasiveness and dissemination, and metastatic colonization [12]. As p21 was turning into an important gene in cancer development, several groups started to think about therapeutic approaches in using p21; one of the first attempts to induce growth arrest via p21 was done in chicken embryo fibroblasts that were transformed by oncogenes [13]. Another pioneer study in T-cell leukemia virus type I-transformed lymphocytes showed p21 playing a role in apoptosis, independent of p53 [14]. p21, continued to be a gene of interest for tumor growth inhibition during the following years [15].

### 1.2. p21 and Cancer Evolution

Controversial aspects of p21 is decided by p21 location and p53 protein condition [16]. p53 (the most mutated protein in pediatric and adult cancer) induces expression of p21, in response to cellular stress, such as DNA damage or oxidative stress. In addition to cell cycle arrest, p21 plays an important role in senescence through p53-dependent and p53-independent pathways [17,18]. p21 also regulates various cellular programs such as apoptosis, DNA damage response, and actin cytoskeleton remodeling. This being said, p21 effect on the evolution of cancer tumors depends largely on the status of the p53 protein in cancer cells [19]. Although p21 induction is p53-dependent in certain conditions such as DNA damage, there are several scenarios in which p21 expression pattern is independent of p53 such as normal tissue development, cellular differentiation, or following serum stimulation [20]. In response to p53 transcription factor activity, p21 induction could lead to tumor growth arrest through inhibition of cyclin-kinase complex, proliferation cell nuclear antigen (PCNA), transcription factors, and coactivators [17]. On the other hand, p21 can direct tumor evolution towards cancer growth through slowing down the accumulation of DNA damage [21]. p21 induction has been shown to be crucial for promoting cancer cell motility and tumorigenesis [22]. Therefore, p21 can be an oncogenic protein or a tumor suppressor, depending on its localization in the cytoplasm or the nucleus, respectively [23,24]. This controversy surrounding p21 roles in cancer evolution makes it more challenging to find the right balance in which p21 function would selectively impede cancer progression. 

### 1.3. LincRNA-p21 and Cancer

mRNA, with only 3–7% of total RNA mass in the cells, has been the focus of most studies so far [25]. Non-coding RNAs, on the other hand, are underappreciated regarding their functional/regulatory activities; although long non-coding RNAs (lncRNA) are a small portion of the total non-coding RNA population (regarding mass and the number of the molecules), they have been under scrutiny in cancer development [26]. LincRNA-p21 (long intergenic noncoding RNA-p21) is located 15 kb upstream of p21 gene and it regulates gene expression both at the transcriptional level as well as post-transcriptionally. Regulated by p53, lincRNA-p21 is an important player in regulating p53 target gene expression through physically interacting with heterogeneous nuclear ribonucleoprotein K (hnRNP K), functioning as a key repressor [27]. LincRNA-p21 regulates cell proliferation, DNA damage response, and apoptosis through its regulatory role in p53 target gene expression [28,29]. LincRNA-21 regulates reprograming through several mechanism; as an example, lincRNA-p21 sustains CpG methylation and/or H3K9me3 at the promoter region of pluripotency genes, causing somatic cell reprogramming prevention [30]. LincRNA-p21 also modulates Warburg affect, hence playing an important role in cancer cell metabolism [31]. It is not surprising that lincRNA-p21 is associated with cancer progression [32]. PANDA (P21-associated noncoding RNA DNA damage-activated) is another lncRNA located 5 kb upstream of p21 gene that regulates proapoptotic genes and senescence through stabilizing p53 tumor suppressor gene [33]. p53 binds to transcription start site of p21 and activates PANDA and p21 transcription in response to DNA damage [34]. 

## 2. p21 as a Target in Cancer Treatment

### 2.1. Gene Editing of p21 in Cancer Cells

Gene editing is mostly used for research purposes; CRISPR (clustered regularly interspaced short palindromic repeats) [35], TALENs (transcription activator-like effector nucleases) [36], ZFNs (zinc finger nucleases) [37], rAAV (recombinant adeno-associated virus) [38], small interference RNA [39], and homologous recombination [40] are gene editing tools that have been used to manipulate gene expression (knocking out, mutating, or silencing). p21 has been altered in in vitro and in vivo models to investigate tumor growth, apoptosis, and cell cycle arrest in cancer cells. Changing p21 expression levels using gene editing can be used as an additive therapy for specific cancers to suppress tumorigenesis phenotypes or to reduce drug resistance. 

p21 has a dual role in cancer [16]. There are several studies that have shown the tumor suppressor function of p21. We have addressed these studies in Table 1. In mouse studies, p21-deficient mice were shown to become susceptible to hematopoietic, epithelial, and endothelial tumor formation [41]; in another study, p21-deficient mice that were injected with colon carcinogen (azoxymethane), developed more putative premalignant lesions [42]. p21 over-expression via adenovirus in prostate cancer cells has been shown to induce apoptosis and reduce tumor volume in mice [43]. In an in vitro study, the same results were shown regarding reduced tumor growth in cervical cancer cells after over-expressing p21 [44]. On the other hand, other studies reported contradictory results, in vitro and in vivo. As for in vitro works, it was shown that C-terminal mutation of p21 in human colon cancer [45] and decreased expression of p21 in human melanoma SK-MEL-110 cells [46] promotes apoptosis. Gene delivery of p21-p27 fusion protein into MCF-7 cell line (adenocarcinoma) was shown to induce apoptosis and suppress proliferation [47]. As for in vivo studies, combined deletions of p21 and p27 in mice models showed aggressive tumor and decreased lifespan [48]. p21 role in cancer gets even more complicated when combined with other CDK inhibitors. Monocytic myeloid-derived suppressor cells (Mo-MDSCs), potent suppressors of tumor immunity with increased p16 and p21 expression, were genetically modified in a recent study; this research concluded that tumor progression was suppressed through inhibiting MO-MDSC accumulation in tumors, following p21 and p16 deletion [49]. In another study, cell proliferation and tumor growth were significantly decreased after introducing p21 and p53 into breast cancer mouse models through injecting nanoparticles, and transfecting cell lines [50]. These studies point out the complexity of p21 in cancer therapy and the importance of combined therapeutic approaches. p21 deficient mouse mammary tumor virus (MMTV)-ras and MMTV-myc transgenic mice models showed different behavior after p21 deletion. Tumors in MMTV-ras/p21^−/−^ mice showed higher S-phase fractions than tumors in MMTV-myc/p21^−/−^ mice. p21 deficiency in these two different mouse models also affected apoptosis levels in different manners. Significant increase in spontaneous apoptosis was reported in MMTV-ras/p21^−/−^ mice tumor, whereas apoptosis levels of MMTV-myc/p21^−/−^ mice tumor did not change significantly. Different oncogene expression patterns caused different cell proliferation profile in p21-deficient mice, pointing out context-dependence function of p21 in cancer progression [51]. p21 was knocked out in NEMO^Δhepa^ (hepatocyte-specific NF-kB Essential Modulator knockout) mice, which are used as hepatocellular carcinoma model. In this study, p21 deficiency was shown to result in higher DNA damage and higher number of hepatocellular carcinoma (HCC) [52]. C57Bl6-^FahDexon5^ mice are liver failure in vivo model with increased risk for liver cancer. Loss of p21 in C57Bl6-^FahDexon5^ mice was shown to cause rapid tumor formation and continuous hepatocyte proliferation [53]. Interestingly, p21 deletion in prostate cancer mice model in another study was shown to lead to less aggressiveness in prostates, lower adenocarcinoma incidence, and protection against prostate tumorigenesis [54]. Adnane et al. showed that p21 deletion in MMTV/v-Ha-ras transgenic mice leads to induced tumor aggressiveness and earlier tumor appearance [55]. In another study p21 was knocked out in Apc1638^+/−^ mice, an intestinal tumor model. Apc1638^+/−^ /p21^−/−^ mice showed altered cell maturation, increased tumor formation and cell proliferation [56].

SOCS1 (suppressor of cytokine signaling 1)-deficiency in mice, treated with hepatocarcinogen diethylnitrosamine (DEN), was shown to cause large and numerous liver tumor nodules. p21 protein level was shown to increase in SOCS1-deficient mouse liver, causing increased resistance to apoptosis and induced proliferation in response to growth factor stimulation. However, these phenotypes disappeared following p21 knockdown [57]. In another in vivo study, Forkhead Box F1 (FoxF1) and Forkhead Box F2 (FoxF2) transcription factor deficiency in mice repealed Alveolar rhabdomyosarcoma (RMS) tumor growth, following increased transcription activity of p21; knocking down p21 in FoxF1 and FoxF2-deficient tumor cells rescued cell cycle progression [58]. p21-deficient prolactinomas (PRL) showed increased tumor migration and proliferation [59]. miR-6734 upregulates p21 expression; in vitro studies of colon cancer have shown that miR-6734 introduction into the cancer cells suppresses phosphorylation of Rb (Retinoblastoma), and cleavage of PARP (Poly (ADP-ribose) polymerase) and caspase 3, causing cancer cell growth inhibition and cell cycle arrest/apoptosis in HCT-116 cells [60]. In another study, p21 deficiency in Eµ-Myc mice, which overexpress c-MYC in B-lymphoid cells, did not change number and survival of pre-leukemic Eμ-Myc B-lymphoid cells [61].

p21 alteration can have a major effect on cancer susceptibility to chemotherapy, radiation, and targeted therapy. As shown in Table 1, p21-deficient cell lines in mentioned in vitro studies showed more sensitivity to cancer drugs (cotreatment of Chk1 (Checkpoint Kinase1 ) inhibitor and platinum based drugs) and irradiation-induced apoptosis [62,63,64]. Cancer cells treated with ginseng [65], imatinib and gefitinib [66], pyrazolo(1,5-a)pyrimidine [67], and microtubule inhibitor (Taxol and vincristine) [68] were showed to have increased apoptosis and cell death when p21 is deficient. Increased cell death with MK1775 checkpoint inhibitor treatment was observed in p21-deficient cells [69]. Increased caspase-3-dependent apoptosis was reported in p21-deficient cells after ionizing radiation [63,64]. In an in vivo study, p21-deficinet mice were exposed to chemical carcinogen (urethane) that predominantly forms lung tumors in mice; tumor formation and tumor multiplicity were increased in these p21-deficient mice [70]. In Table 1, we have illustrated p21-cancer studies with mouse models and cancer cell lines.

### 2.2. Targeting p21 Pathways

**Figure 1:**
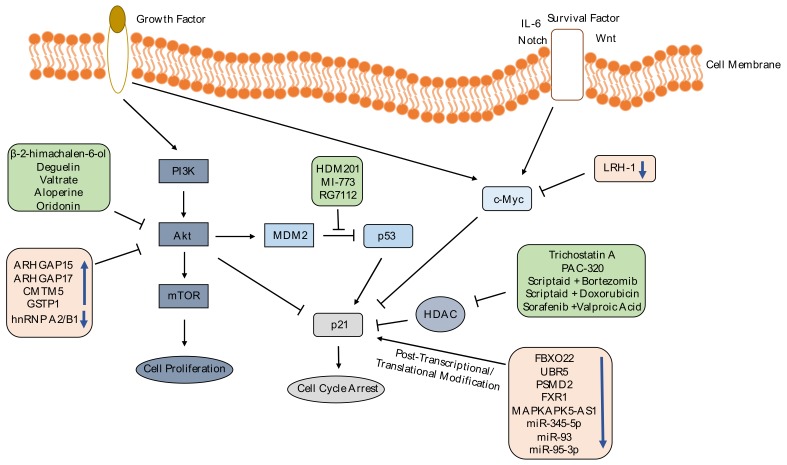
p21 expression induction through PI3K-Akt and c-Myc pathways. In this figure, drugs are shown in green boxes and differentially expressed genes/miRNAs are shown in pink boxes. These differentially expressed genes/miRNAs and drugs positively regulate p21 expression through reducing Akt phosphorylation, inhibiting MDM2-p53 interaction, suppressing c-Myc expression, preventing p21 ubiquitylation, preventing p21 mRNA destabilization, blocking negative regulation of p21 protein, or suppressing histone deacetylation. As a conclusion, increased p21 expression induces cell cycle arrest and decreases cell viability. PI3K: Phosphoinositide 3-Kinase, Akt: Protein kinase B, ARHGAP15: Rho GTPase-Activating Protein 15, ARHGAP17: Rho GTPase Activating Protein 17, c-MYC: cellular Myelocytomatosis Oncogene, CMTM5: CKLF (Chemokine Like Factor) Like MARVEL Transmembrane Domain Containing 5, FBXO22: F-box Only Protein 22, FXR1: Fragile X-related Protein 1, GSTP1: Glutathione S-Transferase Pi 1, HDAC: Histone Deacetylase, hnRNP A2/B1: Heterogeneous Nuclear Ribonucleoprotein A2/B1, IL-6: Interleukin 6, LRH-1: Nuclear Receptor Liver Receptor Homolog-1, MAPKAPK5-AS1: MAPKAPK5 Antisense RNA 1, MDM2: Mouse Double Minute 2, mTOR: Mammalian Target of Rapamycin, PI3K: Phosphoinositide-3-Kinase, PSMD2: 26S Proteasome non-ATPase Regulatory Subunit 2, UBR5: Ubiquitin Protein Ligase E3 Component N-Recognin 5.

As we explained above, targeting p21 gene in cancer treatments is disputable; however, p21 is involved in many pathways that could be targeted instead of p21 itself to overcome the controversy. p21 is regulated in different levels: Transcriptionally, post-transcriptionally and post-translationally [71]; genes or transcription factors that are found in downstream or upstream of p21 in these functional/regulatory pathways could be the target in therapeutic approaches, bypassing the dual role of p21 in cancer development. In Figure 1, we have illustrated several published strategies targeting p21-engaged pathways through gene/miRNA manipulation or drugs intervention. 

#### 2.2.1. Chemicals

There are several studies that investigate the effect of drugs and chemicals on p21 expression in cancer cells; we have shown some of these works in Table 2. Histone deacetylase (HDAC) inhibitors can increase p21 expression, causing cancer cells to undergo cell cycle arrest. Treatment of pancreatic cancer cells with Trichostatin A [72], prostate cancer cells with PAC-320 [73] and ovarian cancer cells with scriptaid, an HDAC inhibitor, combined with bortezomib or doxorubicin [74] enhances the p21 protein expression and induce cell cycle arrest. Treatment of HCC cells with HDAC inhibitors, sorafenib combined with Valproic acid, decreases HCC viability by upregulating p21 expression and other apoptotic genes expression [75]. As mentioned before, p53 tumor suppressor protein induces expression of p21 [76]. p53 is negatively regulated by Mouse Double Minute 2 (MDM2) [77]. p53-MDM2 interaction inhibitors could be used to increase p21 expression for cancer treatment. In literature, there are many inhibitors to disrupt p53-MDM2 interaction such as HDM201 [78], MI-773 [79] and RG7112 [80]. These drugs can restore expression of p53 and p21 and decrease cell viability in tumors. 

Phosphoinositide 3-Kinase-Protein kinase B (PI3K-Akt) signaling pathway is involved in regulation of cell survival, cell cycle progression and cell growth; Akt enhances cell growth and proliferation by phosphorylating p21 [81]. Chemicals that inhibit PI3K-Akt pathway could be used to induce p21 expression and cell cycle arrest in cancer cells. Oridonin, a natural tetracycline diterpenoid, isolated from Rabdosia rubescens, suppresses cell proliferation and increases G2/M cell cycle arrest via inhibiting PI3K-Akt pathway and upregulating p21 and p53 in cancer cells such as in esophageal cancer [82] and oral squamous cell carcinoma cells [83]. Deguelin treatment in human gastric cancer cells [84], β-2-himachalen-6-ol treatment of skin carcinogenesis mouse model [85], Valtrate treatment in human breast cancer cells [86] and Aloperine treatment in prostate cancer cells [87] reduce tumor growth and cell survival by inhibiting PI3K-Akt pathway and upregulating p21 expression. Chemicals that have been shown to induce p21 expression in cancer research are listed in Table 2.

#### 2.2.2. Gene Editing

Manipulating genes that interact with p21 is another alternative to control p21 effect in cancer development. Expression levels of tumor suppressors and oncogenes change in the tumor cells, which leads to cell proliferation and metastasis through PI3K-Akt pathway [88] and c-Myc-p21 interaction [89]. Decreased expression of Rho GTPase-activating protein 15 (ARHGAP15) in colorectal cancer [90], Rho GTPase Activating Protein 17 (ARHGAP17) in cervical cancer cells [91], CKLF (Chemokine Like Factor) like MARVEL (MAL (myelin and lymphocyte) and related proteins for vesicle trafficking and membrane link) transmembrane domain containing 5 (CMTM5) [92] and glutathione S-transferase pi 1 (GSTP1) [93] in HCC have been reported; overexpression of these genes was shown to induce p21 expression by inhibiting PI3K-Akt pathway, leading to suppressed tumor growth and metastasis. Heterogeneous nuclear ribonucleoprotein A2/B1 (hnRNP A2/B1) is overexpressed in cervical cancer. Knocking down hnRNP A2/B1 in in vitro and in vivo studies caused p-AKT expression decrease and p21 expression increase, followed by suppressed proliferation, migration and invasion [94]. c-Myc binds to p21 promoter and negatively regulates p21 expression [95]. Xiao et al. group showed that ectopic expression of liver receptor homolog-1 (LRH-1), which is increased in HCC, leads to upregulation of c-Myc and downregulation of p21. Knocking out LRH-1, decreased cell proliferation and tumor growth [96]. 

#### 2.2.3. Synthetic Lethality in p21-Mutant Cancers 

Synthetic lethality was first introduced by Calvin Bridges to describe when certain non-allelic genes are lethal in combination, while parents with a homozygous mutation are viable; in other words, when two genetic events happen together causing cell death, synthetic lethality occurs [97]. This genetic event becomes useful when undruggable genes are driver mutation in cancer [98]. p21 basal levels determine the sensitivity of cells to combined therapies; in a study on colorectal cancer, synthetic lethality of Chk1 inhibitor in tumors with lower p21 was reported [99]. p21 was shown to be responsible for protecting tumor cells from DNA damage cytotoxic effect after Chk1 inhibition. p21 activated kinase (PAK) proteins, have different functions in different species; these proteins are associated with cancer due to their role in cell survival, cell migration, and proliferation [100]. PAKs are divided into group I (or group A) and group II (or group B), depending on their structure, expression levels, and the tissue type they are expressed in [101,102]. Although PAKs are often not found to be mutated in cancer, PAK dysregulation, especially overexpression, is shown to be correlated with cancer [103]. Although PAK function is mostly found to be important in embryonic development according to in vivo studies [104], there is a positive correlation between PAK expression level and tumor grade, therefore members of both group I and group II PAKs are found to be associated with tumorigenesis [103]. This being said, the most frequently dysregulated PAK members in cancer are PAK1 (group I) and PAK4 (group II) [105]. PAK 1 and PAK4 are found on cancer-induced frequently amplified chromosomal regions, predominantly in breast cancer [106]. PAK1 is a therapeutic target for acute myeloid leukemia (AML); inhibition of PAK1 causes downregulation of MYC oncogene, which leads to AML cell apoptosis [107]. PAK4 was shown to play an important role in prostate cancer cell migration in response to hepatocyte growth factor (HGF); HGF is associated with invasiveness of prostate cancer [108]. In Ras mutant cancers, a critical role for Rac/Pak signaling in promoting mitogen-activated protein kinase (MAPK) activity has been shown [109]. PAK4 overexpression was shown to cause drug resistance and poor survival in gastric cancer patients; knocking down PAK4 induced apoptosis in PAK4 overexpressing gastric cell lines [110]. In a network-based screening method, PAK1 was mapped into many synthetic lethality pathways, such as MAPK signaling, ErbB (Erythroblastic leukemia viral oncogene homologue) signaling, and focal adhesion pathways [111]. PAK1 inhibition in a mouse model of Kras-driven squamous cell carcinoma was shown to suppress tumorigenesis, following loss of Erk and Akt activity. PAKs play an important role in cancer stemness as well, which is briefly explained in “p21 role in stemness” section.

## 3. p21 Regulation

### 3.1. Strict p21 Regulation

Transcriptional, post transcriptional, translational and post translational modifications are regulatory checkpoints for protein expression; these regulations decide localization [112], activity [113], and stability [114] of proteins. p21, induced by p53, binds to dimerization partner, RB-like, E2F and multi-vulval class B (DREAM) complex, and causes downregulation of cell-cycle regulated genes, by stabilizing this complex [115,116]. Since p21 has a crucial role in cell cycle arrest, expression level of p21 should be tightly controlled [117]. Several regulators balance p21 expression in different levels. For example, Rbm24, an RNA-binding protein (RBP), induces expression of p21 mRNA, and therefore p21 protein, by binding to 3´-untranslated region (3´-UTR) of p21 transcript [117]. Fragile X-related protein 1 (FXR1), which is also an RBP, binds to the G-quadruplex RNA structure in mRNA and controls mRNA turnover [118]. FXR1 is overexpressed in head and neck squamous cell carcinoma (HNSCC). Overexpressed FXR1 binds and destabilizes mRNA of p21 and decreases p21 protein expression. Silencing of FXR1 (fragile X-related protein 1) induces expression of p21, which causes the cancer cells to go through senescence [119]. Interaction of telomere repeat binding factor TRF2 (Telomeric repeat-binding factor 2) with p21 promoter G-quadruplex, inhibits p21 expression. It has been shown that induced DNA damage response did not put cancer cells into G2/M cell arrest due to p21 repression through TRF2 binding [120]. Posttranscriptional and translational regulation of p21 is regulated by lncRNA and microRNAs (miRNA). LncRNA MAPKAPK5-AS1 was shown to be upregulated in colorectal cancer (CRC) cells through suppressing p21 expression. Knocking down this lncRNA resulted in apoptosis and inhibition of proliferation in CRC cells [121]. Another study showed that miR-345-5p in prostate cancer (PCa) [122], miR-93 in osteosarcoma cells [123] and miR-95-3p in HCC cells and xenograft mouse models [124] were significantly overexpressed. Overexpressed miRNA inhibits p21 expression which leads to induction of cell growth, proliferation and invasion in cancer cells. 

### 3.2. Targeting p21 Regulators for Therapy

Translational regulation and post translational modifications could be targeted to manipulate p21 function in cancer cells. DEAD-box (D-E-A-D (asp-glu-ala-asp) box) RNA helicase DDX41 (DEAD-box helicase 41) binds to 3’-UTR of p21 mRNA and negatively regulates p21 expression [113]. Galectin-3 (Gal-3), a carbohydrate-binding protein, can also induce p21 expression in human prostate cancer cells with wildtype p53, post-translationally [125]. m5C Methylation by NSUN2 (NOP2 (Nucleolar Protein 2 Homolog)/sun RNA methyltransferase-2) and m6A Methylation by METTL3/METTL14 in 3´UTR of p21 mRNA increase expression of p21 at translation level [126]. Activation of integrated stress response (ISR) kinase GCN2 (General Control Non-depressible 2) through phosphorylation of the eukaryotic translation initiation factor eIF2α leads to induced p21 transcript variant translation with 5’ upstream open reading frames (uORFs) [127]. Two lysine residues of p21 are acetylated by histone acetyltransferase Tip60; Lee et al. showed that deletion of Tip60 causes destabilization of p21 and prevents G1 arrest [128]. Arginine156 in p21 protein is methylated by protein arginine methyltransferase 6 (PRMT6). Arginine156 methylation induces phosphorylation of threonine145 in p21. These post translational modifications increase cytoplasmic localization of p21, which results in more resistant cancer cells to cytotoxic agents [129]. Expression of F-box only protein 22 (FBXO22) in HCC [130], UBR5 (ubiquitin protein ligase E3 component N-recognin 5) in colon cancer [131] and PSMD2 (26S proteasome non-ATPase regulatory subunit 2) in breast cancer [132] is upregulated; these genes regulate p21 stability by mediating ubiquitylation of p21. Following upregulation of mentioned p21 negative-regulators, cell-cycle arrest is prevented, followed by tumor growth and cell proliferation induction. Furthermore, silencing PSMD2 and FBXO22 knockdown were shown to decreased p21 ubiquitylation, inducing p21 expression. p21 is regulated at different levels and it is involved in several pathways. A dominating idea according to most studies on p21 in cancer, is that inducing p21 expression through targeting other genes in p21 cascades could be an effective way to prevent tumor growth and metastasis. These target genes are promising candidates for therapeutic approaches. Table 3 illustrates differentially expressed genes and RNAs that interact with p21. 

## 4. p21 Role in Stemness

### 4.1. p21 Expression and Stemness

p21 controls expansion of human hematopoietic stem cells and cell cycle progression; p21 knockout stem cells were showed to induce cell cycle and stem cell exhaustion, under normal homeostatic condition [133,134]. SOX2 (sex-determining region Y (SRY)-box2) has a role in the maintenance of cancer stem cells (CSCs). In a study on human pancreatic tumors with ectopic SOX2 expression, deletion of SOX2 in cancer cells was shown to cause cell growth inhibition, induced by p21 and p27 [135]. There are several studies showing p21 regulation in CSCs. Han et al. showed that evodiamine specifically targets CSCs in breast cancer cell lines through p53 and p21 function. This study reported accumulation of cancer cells at G2/M phase after evodiamine treatment, and selective cell death of CSCs [136]. In another study, curcumin treatment was shown to sensitize CSC subpopulation to cisplatin chemotherapy by increasing expression of p21; CSC subpopulation in this study was double-positive (CD166^+^/EpCAM^+^) and highly migratory, derived from non-small cell lung cancer (NSCLC) cell lines (A549 and H2170) [137]. Benzyl isothiocyanate (BITC) has chemoprevention effect on breast CSC (bCSC). This inhibition was shown to be negatively regulated by KLF4 (Kruppel-like factor 4) transcription factor and its target gene, p21, in transgenic mouse model of breast cancer [138]. Similar results in another study demonstrated that knocking out p21 in PyMT (polyoma middle tumor-antigen) mammary tumor model inhibits tumor formation/initiation and ALDH1 (aldehyde dehydrogenase 1) activity, all properties of CSCs. This study showed that p21 creates CSC-like phenotype formation by suppressing Wnt/TCF1 (Wingless INT/ Transcription factor T cell factor 1)/Cyclin D1 signaling [139]. 

### 4.2. Non-Coding RNA and Stemness

LincRNA-p21 is downregulated in glioma stem cells (GSCs) following increased expression of Hu antigen R (HuR) through miR-146b-5p downregulation. Overexpression of miR-146b-5p is shown to decrease viability of cell and stemness, increasing apoptosis and radiosensitivity. However, these phenotypes were rescued following lincRNA-p21 knockdown. This study concluded that targeting miR-146b-5p/HuR/lincRNA-p21/β-catenin signaling pathway could be implemented as a co-therapy for glioma cancer patients [140]. In another research, LincRNA-p21 was overexpressed, using adenoviral vector containing miRNA responsive element (MRE) for miR-451. lincRNA-p21 introduction into CSCs, derived from primary colorectal cancer tissues and cell lines, was shown to inhibit β-catenin signaling, causing decreased self-renewal, viability, and glycolysis of these cells. lincRNA-p21 showed no off-target effect on normal liver cells, in vivo [141]. microRNA-7 is a novel tumor-suppressor and its expression is downregulated in prostate cancer cells. Restoration of microRNA-7 suppresses KLF4 (Kruppel-like factor 4 )/PI3K/Akt/p21 pathway in prostate cancer cells, causing decreased tumorigenesis and inhibition in stemness of prostate CSCs [142]. 

### 4.3. PAK and Stemness

p21-activated kinase 3 (PAK3) controls Akt-GSK3β (Glycogen Synthase Kinase 3 beta)-β-catenin signaling in pancreatic cancer cells. PAK3 inhibition leads to suppressed tumorigenesis and CSCs expansion [143]. PAK1 was shown to upregulate CSC markers and cause resistance to 5-fluorouracil (5-FU) chemotherapy, leading to colorectal cancer progression [144]. Expression of PAK4 in the triple-positive (CD24^+^/CD44^+^/EpCAM^+^) subpopulation of pancreatic CSCs is higher than triple- negative (CD24^−^/CD44^−^/EpCAM^−^) cells. PAK4 expression correlates with nuclear accumulation and transcriptional activity of signal transducer and activator of transcription 3 (STAT3). PAK4 silencing in pancreatic cancer (PC) cells was shown to decrease tumorigenesis, increasing PC cells sensitivity to gemcitabine toxicity [145]. PAK4 also affects stemness and cancer resistance to endocrine therapies. Introducing PAK4 small molecule inhibitor (CRT PAKi) and PAK4-targeting siRNAs results in suppressed self-renewal and CSC activity [146].

## 5. Discussion

In this review, we tried to list the available approaches for p21-directed cancer treatment. Not only p21 is involved in many important pathways that are dysregulated in cancer, but the expression of the protein itself is altered in human cancers [17,147]. As p21 function/regulation becomes clearer, the imbalance of p21 in cancer will be easier to address. The dual role of p21 in cancer progression as an oncogene and tumor suppressor [16,148] makes it harder to have one approach for all cancer types; nevertheless, according to the literature, we believe that p21 induction has a synergic effect on other treatments, as it has been shown for several combination therapies, mentioned in this review [149]. On the other hand, looking at studies on cancer stem cells, one could agree that p21 plays an important role in inducing stemness, especially through p21 activated kinases (PAK1, PAK3, and PAK4) [143,144,145,146]. lincRNA-p21, on the other hand, suppresses stem cell expansion and renewal [140,141]. The more we understand p21-associated pathways, the better we can make sense of the contradictory results on p21 role in cancer, as a tumor suppressor or a tumor-promoting protein. It is also very important to realize that p53 status has a great influence on p21 role in cancer development, as p53-independent upregulation of p21, causing DNA replication dysregulation, has been reported in aggressive cancer cells [150]. p21 mutation has been detected in 14% of invasive bladder cancer patients in genome sequencing studies, with half of them also carrying p53 mutation [151]. It is worth noting that p21/p53 double mutant bladder cancer cells have unique Chk1-dependency regarding G2/M cell cycle checkpoint following chemotherapy induced DNA damage [152]; this makes it very crucial to comprehend p21 and p53 status in cancer tumors to choose the most appropriate treatment. For example, gemticabine-Chk1 inhibitor treatment is an effective treatment for p21/p53 double mutant bladder cancer; nevertheless, cancer cells lose their sensitivity to the combined therapy once p21 is restored, pointing out the importance of considering p21-p53 balance in therapeutic approaches [152]. p21 is involved in many aspects of tumorigenesis, and having a deeper understanding of p21 as a double agent protein could be very helpful for adjusting p21 expression levels to control cancer development. Georgakilas et al. has argued that rare p21 mutation rate in cancer might be due to an evolutionary favorable tumor heterogenicity, enforced by p53-independent p21 activity [16]. As we have explained in different contexts, p21 role in promoting or suppressing tumorigenesis depends largely on p53 status. With wildtype p53, p21 acts as guardian of the genome, whereas when p53 is absent or deficient, p21 activity causes genome instability. We discussed several chemical compounds and drugs that could potentially fix p21 imbalance in cancer cells; here again, knowing p53/p21 state is crucial. Another therapeutic scheme could be downregulation of p21, combined with activated p53; for example, adenovirus-expressing p53 combined with p21-targetting microRNA [153] or p53 induction through MDM2 suppression combined with adenovirus-mediated p21 downregulation [154]. Aside from p53 condition, p21 localization also should be taken into account when considering p21 therapeutic approaches since cytoplasmic p21 favors antiapoptotic activities, and nuclear p21 is linked to cell cycle arrest [155,156]. According to the literature, the absence or presence of p21 influences sensitivity to chemotherapy or radiotherapy, largely due to p21 involvement in important signaling pathways such as PI3K-Akt and c-Myc. Although p21 status in cancer development and progression remains controversial, p21 is an important contributor in cancer aggressiveness/stemness, drug resistance, and invasiveness; p21 mutation being rare in cancer should not distract us from considering this protein’s influence in the fate of cancer cells. 

## Figures and Tables

**Table 1:** p21 deficient in vivo and in vitro models used in p21-cancer studies.

Phenotype of Mice	Cancer Model	Description	Reference
p21^−/−^	Hematopoietic tumor Epithelial tumor Endothelial tumor	Susceptible to spontaneous tumors development	[41]
p21^−/−^	Colon cancer	Increased putative premalignant lesions development	[42]
p21^−/−^ p16^−/−^	Lewis lung carcinoma	Inhibition of MO-MDSC, accumulation in tumors, and suppression of tumor progression	[49]
p21^−/−^ p27^−/−^	Pituitary adenomas, Pheochromocytomas Thyroid adenomas	Aggressive tumor and decreased lifespan	[48]
p21^−/−^	HCT116	Combination of Chk1 inhibitors and cisplatin treatment enhances cancer cell vulnerability	[62]
p21^−/−^	HCT116	Caspase-9 and caspase-3 dependent apoptosis after ionizing radiation	[63]
p21^−/−^	HCT116	Enhanced caspase-3-dependent apoptosis after irradiation	[64]
p21^−/−^	HCT116	Increased apoptosis and cell death with treatment of ginseng, imatinib and gefitinib, pyrazolo(1,5-a)pyrimidine, microtubule inhibitors, MK1775 checkpoint inhibitor	[65] [66] [67] [68] [69]
p21^−/−^	MMTV-ras	Higher S-phase fractions, increased spontaneous apoptosis	[51]
p21^−/−^	MMTV-myc	Lower S-phase fractions, no effect on apoptosis	[51]
NEMO^Δhepa^/p21^−/−^	HCC	Higher DNA damage and higher number of HCC	[52]
p21^−/−^	Lung tumors	Accelerated tumor onset, increased tumor multiplicity	[70]
Fah^−/−^/p21^−/−^	HCC	Rapid tumor formation, continuous hepatocyte proliferation	[53]
p21^−/−^	Prostate cancer	Less aggressiveness in prostates, lower adenocarcinoma incidence and prostate tumorigenesis	[54]
p21^−/−^	MMTV/v-Ha-ras	Increase in aggressiveness and tumor multiplicity, earlier tumor appearance	[55]
Apc1638^+/−^ / p21^−/−^	Intestinal tumor	Increased tumor formation	[56]
p21^−/−^	miR-6734 expressing HCT-116 cells	Tumor growth and not induction of cell cycle arrest and apoptosis, phosphorylation of Rb and cleavage of PARP and caspase 3	[60]
(SOCS)1^−/−^ /p21^−/−^	HCC	No increased resistance to apoptosis and no increased proliferation to growth factor stimulation	[57]
FoxF1^−/−^ / FoxF2^−/−^ / p21^−/−^	RMS	Restored cell cycle progression	[58]
p21^−/−^	c-MYC-driven lymphoma	No change on the number and survival of pre-leukemic Eμ-Myc B-lymphoid cells	[61]
p21^−/−^	PRL	Increase in migration and tumor formation	[59]

Chk1: Checkpoint kinase 1, c-MYC: cellular Myelocytomatosis oncogene, FoxF1: Forkhead Box F1, FoxF2: Forkhead Box F2, HCC: Hepatocellular carcinoma, HCT116: Human colon adenocarcinoma cell lines, MMTV: mouse mammary tumor virus, Mo-MDSCs: Monocytic myeloid-derived suppressor cells, NEMO: NF-kB Essential Modulator, PARP: (Poly (ADP-ribose) polymerase, PRL: Prolactinomas, RMS: Rhabdomyosarcoma, (SOCS)1: Suppressor of cytokine signaling.

**Table 2:** Drugs/Chemicals that could be used to upregulate p21 expression; target pathways and studied cancer types are shown below.

Drug/Chemical	Target/Pathway	Cancer Type	Reference
β-2-himachalen-6-ol	Akt/PI3K-Akt	Skin Carcinogenesis	[85]
Valtrate	Akt/PI3K-Akt	Breast Cancer	[86]
Deguelin	Akt/PI3K-Akt	Gastric Cancer	[84]
Aloperine	Akt/PI3K-Akt	Prostate Cancer	[87]
Oridonin Oridonin	Akt/PI3K-Akt Akt/PI3K-Akt	Esophageal Cancer Oral Squamous Cell Carcinoma	[82] [83]
HDM201	MDM2-p53 binding	p53 Wild- Type Cancers	[78]
MI-773	MDM2-p53 binding	Mucoepidermoid Carcinoma	[79]
RG7112	MDM2-p53 binding	Neuroblastoma Cancer	[80]
Trichostatin A	HDAC	Pancreatic Cancer	[72]
PAC-320	HDAC	Prostate Cancer	[73]
Scriptaid+Bortezomib	HDAC	Ovarian Cancer	[74]
Scriptaid + Doxorubicin	HDAC	Ovarian Cancer	[74]
Sorafenib+Valproic Acid	HDAC	HCC	[75]

PI3K: Phosphoinositide 3-Kinase, Akt: Protein Kinase B, MDM2: Mouse Double Minute 2, HDAC: Histone Deacetylase, HCC: Hepatocellular Carcinoma.

**Table 3:** Differentially expressed genes and RNAs in different cancer types and their target pathways are shown below.

Gene/Protein/lncRNA/miRNA	Expression in Cancer Cells	Target/Pathway	Cancer Type	Reference
ARHGAP15	Downregulated	Akt/PI3K-Akt	CRC	[90]
ARHGAP17	Downregulated	Akt/PI3K-Akt	Cervical Cancer	[91]
CMTM5	Downregulated	Akt/PI3K-Akt	HCC	[92]
GSTP1	Downregulated	Akt/PI3K-Akt	HCC	[93]
hnRNP A2/B1	Overexpressed	Akt/PI3K-Akt	Cervical Cancer	[94]
LRH-1	Overexpressed	c-Myc/c-Myc-p21	HCC	[96]
FBXO22	Overexpressed	p21/ubiquitylation	HCC	[130]
UBR5	Overexpressed	p21/ubiquitylation	Colon Cancer	[131]
PSMD2 FXR1 MAPKAPK5-AS1 miR-345-5p miR-93 miR-95-3p	Overexpressed Overexpressed Overexpressed Overexpressed Overexpressed Overexpressed	p21/ubiquitylation p21/Posttranscriptional p21/Posttranscriptional p21/Posttranscriptional p21/Translational p21/Posttranscriptional	Breast Cancer HNSCC CRC Prostate Cancer Osteosarcoma Cells HCC	[132] [119] [121] [122] [123] [124]

ARHGAP15: Rho GTPase-activating protein 15, ARHGAP17: Rho GTPase Activating Protein 17, CMTM5: CKLF(Chemokine Like Factor) Like MARVEL Transmembrane Domain Containing 5, CRC: Colorectal Cancer Cells, FBXO22: F-box Only Protein 22, FXR1: fragile X-related protein 1, GSTP1: Glutathione S-Transferase Pi 1, HCC: Hepatocellular Carcinoma, hnRNP A2/B1: Heterogeneous Nuclear Ribonucleoprotein A2/B1, HNSCC: Head and Neck Squamous Cell Carcinoma, LRH-1: Nuclear Receptor Liver Receptor Homolog-1, MAPKAPK5-AS1: MAPKAPK5 Antisense RNA 1, PI3K: Phosphoinositide 3-Kinase, Akt: Protein Kinase B, MDM2: Mouse Double Minute 2, PSMD2: 26S Proteasome non-ATPase Regulatory Subunit 2, UBR5: Ubiquitin Protein Ligase E3 Component N-recognin 5.
